# Structural adaptation of extreme halophilic proteins through decrease of conserved hydrophobic contact surface

**DOI:** 10.1186/1472-6807-11-50

**Published:** 2011-12-22

**Authors:** Alessandro Siglioccolo, Alessandro Paiardini, Maria Piscitelli, Stefano Pascarella

**Affiliations:** 1 Dipartimento di Scienze Biochimiche "A. Rossi Fanelli", Università di Roma La Sapienza, 00185 Roma, Italy

## Abstract

**Background:**

Halophiles are extremophilic microorganisms growing optimally at high salt concentrations. There are two strategies used by halophiles to maintain proper osmotic pressure in their cytoplasm: accumulation of molar concentrations of potassium and chloride with extensive adaptation of the intracellular macromolecules ("salt-in" strategy) or biosynthesis and/or accumulation of organic osmotic solutes ("osmolyte" strategy). Our work was aimed at contributing to the understanding of the shared molecular mechanisms of protein haloadaptation through a detailed and systematic comparison of a sample of several three-dimensional structures of halophilic and non-halophilic proteins. Structural differences observed between the "salt-in" and the mesophilic homologous proteins were contrasted to those observed between the "osmolyte" and mesophilic pairs.

**Results:**

The results suggest that haloadaptation strategy in the presence of molar salt concentration, but not of osmolytes, necessitates a weakening of the hydrophobic interactions, in particular at the level of conserved hydrophobic contacts. Weakening of these interactions counterbalances their strengthening by the presence of salts in solution and may help the structure preventing aggregation and/or loss of function in hypersaline environments.

**Conclusions:**

Considering the significant increase of biotechnology applications of halophiles, the understanding of halophilicity can provide the theoretical basis for the engineering of proteins of great interest because stable at concentrations of salts that cause the denaturation or aggregation of the majority of macromolecules.

## Background

Organisms thriving in "extreme environments", such as thermophiles, alkalophiles, acidophiles, halophiles, piezophiles and psychrophiles, have drawn much interest in the scientific community because of the molecular adaptation they underwent during evolution and for their biotechnological potential [1-4]. The environmental challenges that extremophilic organisms have to face necessitate, besides other physiological modifications, biosynthesis of macromolecules stable and active at environmental physical-chemical extreme conditions. These macromolecules display clearly distinguished features when compared to the macromolecules from microorganisms found in the "normal" (mesophilic) environments [5].

Halophilic microorganisms are salt-loving extremophilic organisms that grow optimally at high salt concentrations. They were found [6] mainly in marine salterns and hypersaline lakes, such as the Great Salt Lake and the Dead Sea. A survey of the salt requirements in the microbial world shows a continuum of properties which makes it very difficult to define by sharp limits what a halophilic microorganism is. The accepted view [6] distinguishes the halophilic organisms in: extreme halophiles (growing best in media containing 2.5-5.2 M salt), borderline extreme halophiles (growing best is media containing 1.5-4.0 M salt), moderate halophiles (growing best in media containing 0.5-2.5 M salt), and halotolerant microorganisms that do not show an absolute requirement for salt for growth but grow well up to often very high salt concentrations (considered extremely halotolerant if the growth range extends above 2.5 M salt).

Distribution of halophilic microorganisms within the tree of life shows that they are widespread in the bacterial and *Archaea *kingdoms. Eukaryotic halophilic microorganisms, such as fungi and algae, are also known [7].

All halophilic microorganisms share a basic property: their cytoplasm must be at least isoosmotic with their surrounding medium. There are two strategies used by halophilic microorganisms to maintain proper osmotic pressure in their cytoplasm. The first involves accumulation of molar concentrations of potassium and chloride. This strategy requires extensive adaptation of the intracellular enzymatic machinery to the presence of salts, as the proteins should maintain their proper conformation and activity at near-saturating salt concentrations. The proteome of such organisms is highly acidic, and most proteins denature when suspended in low salt. Generally, the microorganisms relying on such "salt-in" strategy are obligate halophilic *Archaea*. The other strategy of haloadaptation is based on the biosynthesis and/or accumulation of organic osmotic solutes (osmolytes) such as ectoin, glycine betaine or others [8]. Cells relying on this strategy exclude salts from their cytoplasm as much as possible. The high concentrations of organic "compatible" solutes do not greatly interfere with normal enzymatic activity. Fewer adaptations of the cells' proteome are therefore needed. Such organisms can often adapt to a broad salt concentration range [6].

Halophilic proteins from "salt-in" organisms are distinguished from their non-halophilic homologous proteins by exhibiting remarkable instability in low salt concentration and by maintaining soluble and active conformations in an environment generally detrimental to other proteins. Indeed, hypersaline conditions favor protein aggregation and collapse, interfere with the electrostatic interactions between protein residues, and are responsible for a general decrease in the availability of water molecules [9]. Halophilic proteins, rather than being unfolded by these conditions, appear to be dependent on the presence of salts. In recent years, detailed investigations have tried to unveil the relationships between structure and stability in halophilic proteins [10]. In particular, these studies suggested that the halophilic proteins bind significant amounts of salt and water in solvent conditions similar to their physiological environment. The peculiar ability of halophilic proteins to bind large amount of salts is largely dependent on the number of acidic amino acids on protein surface [11-17]. The role of electrostatic interactions in the stability and folding of halophilic proteins has been investigated and recognized as a key determinant of haloadaptation [18,19]. Moreover, it was observed that halophilic proteins are characterized by a general decrease in hydrophobic amino acid frequency and a greater propensity to form random-coil structures, rather than α-helices [15]. Indeed, protein folding and adequate stability of the native structure in a hypersaline environment may require evolutionary modulation of the hydrophobic interactions occurring at the protein core. Most of the studies carried out to unveil the structural characteristics of halophilic proteins, were based on sequence comparison at proteome and genome levels or were focused onto single or few protein families. These analyses provided undoubtedly valuable indications on the biophysical and biochemical properties of the halophilic proteins. However, scrutiny of proteome and genomic sequences may not unravel subtle differences at the three-dimensional structural level while structural analysis of a single or few protein families may lack sufficient generalization. For these reasons we report in this work a systematic comparison between the available three-dimensional structures of halophilic enzymes deposited in the data banks and the structure of one of their homologues, to investigate the differences possibly related to shared strategies of structural adaptation to high salt environments. Use of three-dimensional structure made it possible to investigate subtle modifications of the surface and hydrophobic core of the halophilic proteins especially at the level of conserved hydrophobic contacts [20].

## Results

### Data set

A set of 15 halophilic enzymes, 9 of which from "extreme" halophiles and 6 from "halotolerant" organisms, was collected along with their non-halophilic structural homologues (Table 1). Among the halophilic enzymes, 8 come from *Archaea *that adopt the "salt-in" strategy (SALTIN), and 7 from *Eubacteria *that adopt the "osmolytes" strategy (OSMOL). In this paper, the entire analysis was carried out considering the two groups separately.

**Table 1:** Data set.

	PDB^a)^	Q^b)^	Organism^c)^	[NaCl]^d)^	Survival^e)^	Res(Å)^f)^	Name^g)^	PDB^a)^	Q^b)^	Organism^c)^	Res(Å)^f)^	Sequence identity(%)^h)^
1	1DOI	1	*Haloarcula marismortui *(A)	3.4-3.9M	Salt-in	1.90	Ferredoxin	1FXA	1	*Anabaena sp*. (B)	2.50	51%
2	1NWZ	1	*Halorhodospira halophila *(B)	1.5-5.1M	Osmolytes	0.82	Photoreceptor	1MZU	1	*Rhospirillum centenum *(B)	2.00	46%
3	1TJO	12	*Halobacterium salinarum *(A)	3.9M	Salt-in	1.60	DNA-protecting protein	2VXX	12	*Thermosinechococcus elongatus *(B)	2.40	36%
4	2B5W	2	*Haloferax mediterranei *(A)	3.4-4.3M	Salt-in	1.60	Glucose dehydrogenase	2CD9	4	*Sulfolobus solfatarius *(A)	1.80	30%
5	2CC6	12	*Halobacterium salinarum *(A)	3.9M	Salt-in	1.27	Dodecin	2V18	12	*Thermus thermophilus *(B)	2.59	42%
6	3IBM	2	*Halorhodospira halophila *(B)	1.5-5.1M	Osmolytes	2.00	Cupin 2 domain-containing protein	3KGZ	2	*Rhodopseudomonas palustris *(B)	1.85	44%
7	1ITK	2	*Haloarcula marismortui *(A)	3.4-3.9M	Salt-in	2.00	Catalase-peroxidase	2FXG	2	*Burkolderia pseudomallei *(B)	2.00	60%
8	2AZ3	6	*Halobacterium salinarum *(A)	3.9M	Salt-in	2.20	Nucleoside diphosphate kinase	3B54	6	*Saccharomyces cerevisiae *(B)	3.10	54%
9	2J5K	4	*Haloarcula marismortui *(A)	3.4-3.9M	Salt-in	1.95	Malate dehydrogenase	1Y6J	2	*Clostridium thermocellum *(B)	3.01	33%
10	1CNO	2	*Marinobacter hydrocarbonoclasticus *(B)	0.6-0.85M	Osmolytes	2.20	Cytochrome *c*_552_	1ETP	1	*Pseudomonas stutzeri *(B)	2.20	47%
11	1NML	1	*Marinobacter hydrocarbonoclasticus *(B)	0.6-0.85M	Osmolytes	2.20	Cytochrome *c *peroxidase	3HQ6	2	*Geobacter sulfurreducens *(B)	2.20	64%
12	2VPN	1	*Halomonas elongata *(B)	0.5-1.4 max 5.5M	Osmolytes	1.55	Prisplasmic ectoin-binding protein	3FXB	1	*Ruegeria pomeroyi *(B)	2.90	62%
13	3IFV	3	*Haloferax volcanii *(A)	1.7M	Salt-in	2.00	Proliferating cell nuclear antigen	1RWZ	3	*Archaeglobus fulgidus *(A)	1.80	36%
14	3IGN	1	*Marinobacter aquaeloi vt8 *(B)	0.6-0.85M	Osmolytes	1.83	GGDEF domain	3I5C	2	*Pseudomonas aeruginosa pao1 *(B)	1.94	40%
15	3BSM	8	*Chromohalobacter salexigens *(B)	1.2-1.7M	Osmolytes	2.20	D-mannonate dehydratase	2QJJ	8	*Novosphingobium aromaticivorans *(B)	1.80	66%

List of protein pairs utilized in the work. Boldfaced PDB codes indicate extreme halophiles.

^a) ^Protein Data Bank code

^b) ^Number of monomers in the biological unit

^c) ^Source organisms. (A) = *Archaea*; (B) = Bacteria

^d) ^Optimal range of NaCl concentration for growth. The first 9 lines contain structures from extreme halophiles; the remaining 6 lines report moderate halophiles.

^e) ^The survival strategy adopted by corresponding organism: "salt-in" or "osmolytes" strategy

^f) ^Crystallographic resolution

^g) ^Protein name as reported in the Protein Data Bank

^h) ^Sequence percentage of identity between protein pairs

Four pairs of halophilic enzymes (PDB ID: 2B5W, 2J5K, 1NML, 3IGN) have a quaternary structure different from their non-halophilic homologs (Table 1) and therefore were excluded from the calculations involving proteins in their quaternary structure, such as calculation of the electrostatic potential surface. The pair cytochrome c_552 _from *Marinobacter hydrocarbonoclasticus *(PDB ID: 1CNO) and from *Pseudomonas stutzeri *(PDB ID: 1ETP) has been included in the analysis, for the reasons explained in Methods section.

### Accessible surface area and electrostatic potential

To calculate the relative solvent accessibility (ASA) and the magnitude of the relative polar and apolar components, protein structures were used in their quaternary form. The surface polar area formed by oxygen and nitrogen atoms was calculated. The area subset formed only by side-chain oxygen or nitrogen atoms (namely, excluding atoms belonging to the polypeptide backbone) was also considered.

In SALTIN enzymes, the fractional apolar component of the relative solvent accessible surface is significantly smaller than the corresponding area in non-halophilic counterparts (*Δ*ApA in Table 2). The polar area formed by oxygen atoms is significantly larger in halophilic enzymes; the same result is obtained when the analysis is repeated excluding backbone oxygen atoms and including only side-chain oxygen atoms. Likewise, the polar area formed by nitrogen atoms is significantly smaller than the corresponding area in non-halophilic counterparts; the same result is obtained when the analysis is repeated without backbone nitrogen atoms and including only side-chain nitrogen atoms. These differences are reflected in the surface electrostatic potential, which is significantly more negative in halophilic enzymes than in the non-halophilic counterparts (Table 3). In OSMOL sample, the apolar component of the relative solvent accessibility was also significantly smaller than the corresponding area in non-halophilic counterparts (Table 2); however, the decreased surface is caused solely by the increase of the polar area formed by side-chain oxygen atoms. In fact, there is no significant difference in the areas formed by oxygen atoms or nitrogen atoms. The analysis was repeated considering only the subset of residues identically conserved between the halophilic and the corresponding non-halophilc homologue in the SALTIN and OSMOL samples. In this case, no significant difference was seen in the exposed areas (see additional file 1).

**Table 2:** *Δ*ASA in the SALTIN and OSMOL samples.

SALTIN HALOPHILES	NON HALOPHILES	*Δ*ApA^a)^	*Δ*Tot O^b)^	*Δ*Sc O^c)^	*Δ*Tot N^d)^	*Δ*Sc N^e)^	OSMOL HALOPHILES	NON HALOPHILES	*Δ*ApA^a)^	*Δ*Tot O^b)^	*Δ*Sc O^c)^	*Δ*Tot N^d)^	*Δ*Sc N^e)^
1DOI	1FXA	-0.07	0.06	-0.08	0.01	0.01	1NWZ	1MZU	-0.05	0.08	0.05	-0.03	-0.02
1TJO	2VXX	-0.09	0.14	0.15	-0.04	-0.04	3IBM	3KGZ	-0.07	0.02	0.02	0.04	0.04
2CC6	2V18	-0.07	0.17	0.15	-0.10	-0.09	1CNO	1ETP	-0.02	0.00	0.01	0.01	0.02
1ITK	2FXG	-0.10	0.13	0.14	-0.03	-0.03	2VPN	3FXB	-0.06	0.06	0.05	-0.01	-0.00
2AZ3	3B54	-0.02	0.09	0.08	-0.06	-0.06	3BSM	2QJJ	0.00	0.00	0.00	0.01	0.01
3IFV	1RWZ	-0.03	0.07	0.08	-0.04	-0.03							

Total^f)^		-0.38	0.66	0.52	-0.28	-0.25			-0.21	0.16	0.14	0.02	0.05
Average^g)^		-0.06	0.11	0.90	-0.05	-0.04			-0.04	0.03	0.03	0.00	0.01
t-test^h)^		**0.00**	**0.00**	**0.05**	**0.02**	**0.03**			**0.03**	0.11	0.07	0.71	0.42
Wilcoxon^h)^		**0.03**	**0.03**	**0.05**	**0.05**	**0.05**			**0.04**	0.14	**0.04**	0.50	0.50

Differences of fractional accessibility surface area (*Δ*ASA) in the SALTIN and OSMOL samples for different class of atoms. The differences are between the surface areas calculated in the halophilic protein and the corresponding areas in the non-halophilic counterpart. The calculations were performed considering the proteins in their quaternary structure.

a) Apolar *Δ*ASA difference between fractional apolar exposed areas of the halophilic protein and the corresponding non-halophilic homolog

b) Oxygen atom fractional *Δ*ASA

c) Side-chain oxygen atom fractional *Δ*ASA

d) Nitrogen atom fractional *Δ*ASA

e) Side-chain nitrogen fractional *Δ*ASA

f) Total fractional *Δ*ASA

g) Average fractional *Δ*ASA

h) Boldfaced and underlined *p*-values indicate significant or possible trend, respectively

**Table 3:** *Δ*AAP for SALTIN and OSMOL samples.

SALTIN HALOPHILES	NON HALOPHILES	*Δ*AAP	OSMOL HALOPHILES	NON HALOPHILES	*Δ*AAP
1DOI	1FXA	-5.96	1NWZ	1MZU	-2.91
1TJO	2VXX	-76.92	3IBM	3KGZ	-6.85
2CC6	2V18	-49.24	1CNO	1ETP	4.83
1ITK	2FXG	-25.62	2VPN	3FXB	-2.89
2AZ3	3B54	-32.92	3BSM	2QJJ	0.06
3IFV	1RWZ	-17.59			

Total^a)^		-208.26			-7.75
Average^b)^		-34.71			-1.55
t-test^c)^		**0.02**			0.47
Wilcoxon^c)^		**0.03**			0.50

Differences of average atomic potential (*Δ*AAP) for SALTIN and OSMOL samples. Units are kT/e.

a) Total *Δ*AAP

b) Average *Δ*AAP

c) Boldfaced digits indicate significant *p*-values

Surface electrostatic potential displays no significant difference although, in 3 out of 5 pairs, it is more negative in the OSMOL halophilic proteins (Table 3).

### Apolar contact area

The apolar contact area (ACA) between residues was calculated for three different structural regions namely the core, the interface between the monomers and the protein surface. Residues were assigned to the various structural regions according to the alternative accessibility thresholds described in the Methods section.

**Figure 1:**
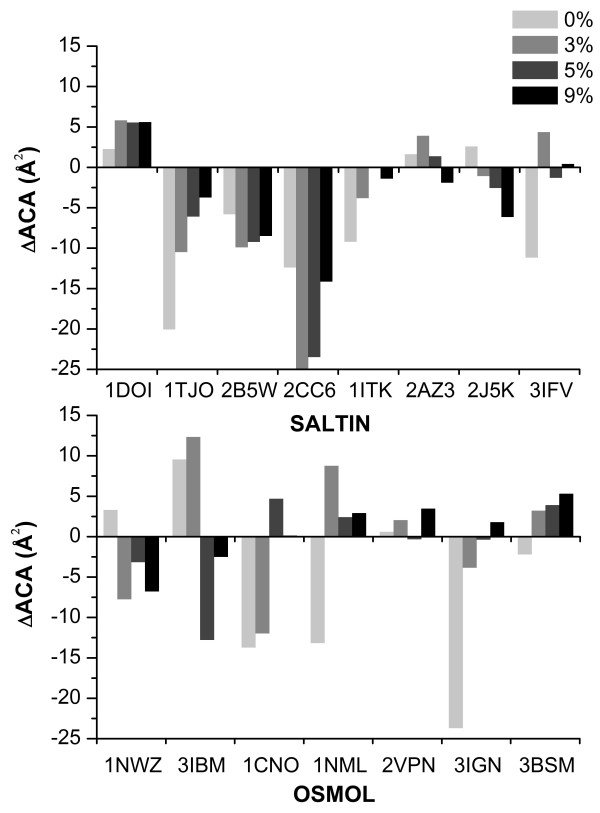
**core *Δ*ACA**. Histograms reporting the core *Δ*ACA in the SALTIN (upper panel) and OSMOL (lower panel) samples at different solvent accessibility thresholds (thresholds and relative grey codes are reported in the box in the upper right corner of the figure). Residues with accessibility less than the thresholds are considered during calculation of the differences. PDB IDs of the halophilic protein are reported on the horizontal axes.

ACA in the core of SALTIN halophilic proteins is not significantly different, according to the statistical tests, from the ACA of their homologous counterparts. However, it is consistently smaller (Figure 1). This trend is more pronounced when the core contains only residues at 0% accessibility. At variance, OSMOL enzymes have an ACA that is globally comparable with that of non-halophilic counterparts (Figure 1), even at 0% accessibility threshold.

Likewise, ACA in the SALTIN monomer interface is not significantly dissimilar from the ACA of their non-halophilic counterparts. However, even in this case, all the *Δ*ACAs were negative except for the pair 3IFV-1RWZ. The analysis at interface for OSMOL enzymes is reduced to two pairs and is therefore of no statistical interest, however, the ACA formed by interfacial residues in halophiles is comparable to that of non-halophilic organisms.

**Figure 2:**
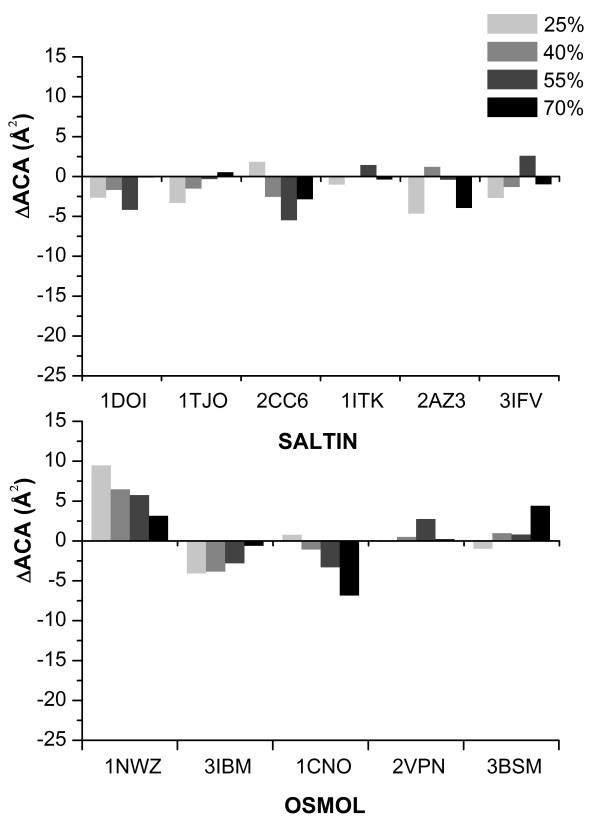
**surface *Δ*ACA**. Histograms reporting the surface *Δ*ACA in the SALTIN (upper panel) and OSMOL (lower panel) samples at different solvent accessibility thresholds (thresholds and relative grey codes are reported in the box in the upper right corner of the figure). Residues with accessibility greater than the thresholds are considered during the calculation of the differences. PDB codes of the halophilic protein are reported on the horizontal axes.

The surface ACA in SALTIN and OSMOL halophilic enzymes is not significantly different from the corresponding surface in the non-halophilic counterparts (Figure 2).

Differences in apolar area buried upon folding (*ΔΔ*ApA_U-F_) between each halophilic and non-halophilic pair, have been calculated. According to the Wilcoxon and the *t*-test, none of the average values were significantly different from 0. Distribution of the differences in the SALTIN and OSMOL samples are reported in additional file 2.

### Conserved hydrophobic contact area

All 15 structures collected from halophilic organisms have been structurally aligned with the non-halophilic counterparts, and the structurally conserved regions (SCR) were calculated. The conserved contacts between hydrophobic residues (CHC) were identified for each pair of structures, and were selected only those formed by evolutionary conserved residues. Then for each CHC, the apolar contact area formed by the pair of residues was calculated. The number of CHCs selected varies for each enzymatic family depending on the relative degree of conservation of residues in that family (Table 4).

**Table 4:** Number of CHCs found in the two samples with the corresponding *Δ*ACA.

SALTIN HALOPHILE	Seq. lenght	NON HALOPHILE	Seq. lenght	Sequence identity(%)	No. CHC	OSMOL HALOPHILE	Seq. lenght	NON HALOPHILE	Seq. lenght	Sequence identity(%)	No. CHC
1DOI	128	1FXA	98	51	50	1NWZ	125	1MZU	129	46	55
1TJO	182	2VXX	192	36	36	1CNO	87	1ETP	190	44	26
2B5W	357	2CD9	366	30	154	1NML	326	3HQ6	345	47	157
2CC6	68	2V18	68	42	33	2VPN	316	3FXB	326	64	132
1ITK	731	2FXG	748	60	514	3IGN	177	3I5C	206	62	63
2AZ3	164	3B54	161	54	71	3BSM	413	2QJJ	402	40	187
2J5K	304	1Y6J	318	33	101	3IBM	167	3KGZ	156	66	65
3IFV	247	1RWZ	245	36	104						

Total^a)^					329.33						58.56
Average^b)^					-0.31						-0.08
t-test^c)^					**0.03**						0.52
Wilcoxon^c)^					**0.01**						0.63

Number of conserved hydrophobic contacts (CHCs) found in the two samples and the corresponding overall difference of apolar contact area (*Δ*ACA)

a) Total *Δ*ACA in the CHCs

b) Average *Δ*ACA in the CHCs

c) Boldfaced digits indicate significant *p*-values

In SALTIN sample the average ACA in CHCs is significantly smaller in halophilic enzymes than non-halophilic counterparts (Table 4). At variance, OSMOL sample displays no significant difference. It has been also tested whether the differences of the CHC areas in the halophilic and non-halophilic counterparts correlated with sequence identity. No correlation was found (see additional file 3).

Statistics on the preferred amino acid exchanges at the SALTIN CHCs indicate that Ile → Val is the most frequent replacement found in the direction mesophile **→ **halophile with a *Z*-score equal to 4.34. At variance, the most frequent exchange in the OSMOL CHCs is Leu → Ile with Z-score 3.74.

## Discussion

Halophilic proteins have been extensively studied since a long time [10] and very much is known about their distinguishing characteristics, although several aspects not sufficiently elucidated of their molecular adaptation mechanisms still remain. Indeed, most of the published studies relied on comparative analyses of the characteristics of halophilic proteome and genomic sequences that provided general overview of the molecular signature of haloadaptation [15] but may not unravel subtle differences at the three-dimensional structural level. Likewise, three-dimensional structural comparisons were generally carried out within a single or a few protein families which may not be sufficiently representative of the halophilic proteome. Therefore, our work was aimed at contributing to the understanding of the shared molecular mechanisms of protein haloadaptation through a detailed and systematic comparison of a larger sample of several three-dimensional structures of halophilic and non-halophilic proteins now available in the databanks. This approach highlights the adaptation strategies shared by the extreme halophilic proteins, besides the variations related to the peculiarities of each single family. Indeed, the extent to which individual proteins adapt to halophilic conditions varies, presumably due to their diverse characteristics and roles within the cell. For example, the malate dehydrogenase from *Salinibacter ruber *recently characterized [21] shares characteristics of a haloadapted archaeal enzyme and of non-haloadapted enzymes from other eubacterial species. Moreover, a proliferating cell nuclear antigen from *Haloferax volcanii *[16] displayed an unexpectedly low number of ion pairs at the monomer-monomer interface.

Proteins were taken both from microorganisms (mostly extreme halophilic *Archaea*) that utilize the "salt-in" strategy to cope with the strong environmental osmotic pressure, and from halophilic microorganisms using the intracellular accumulation of osmolytes and low cytoplasmatic salt concentration to counterbalance the external osmotic pressure. Proteins synthesized by the "salt-in" organisms are surrounded by a high-salt concentration environment and are in contact with it. At variance, intracellular proteins from the "osmolyte" cells are surrounded by a high concentration solution of compatible solutes which apparently do not interfere significantly with protein solubility, stability and activity [22]. Structural differences observed between the "salt-in" and the mesophilic homologous proteins (this set was referred to as SALTIN sample) were contrasted to those observed between the "osmolyte" and mesophilic pairs (here called OSMOL sample). The reasons for this comparative approach is twofold: the OSMOL sample was the reference sample against which to test the structural differences observed in the SALTIN sample; at the same time we verified the presence of possible molecular adaptations in the OSMOL proteins due to their immersion into the osmolyte solution. As a further filter to eliminate possible noise due to non-normality distribution or paucity of data, two statistical tests (the parametric *t*-test and the non-parametric Wilcoxon test) were used to assess the significance of observed differences of structural parameters. Only differences defined significant according to both tests were deemed *bona fide *significant signals.

Starting from the surface features of the halophilic proteins, we demonstrated a significant reduction of apolar surface in the SALTIN sample and a parallel increase of the polar area formed by the side chain oxygen atoms, while the nitrogen side chain atoms lessen their contribution to the surface. These results reflect the well-known increase of surficial aspartate and glutamate residue frequency combined to the decrease of lysine residues [15,23]. It should be mentioned indeed that a reduction of hydrophobic exposed surface in the glucose dehydrogenase from *Haloferax mediterranei *has been demonstrated [24]. In this case however, reduction of apolar surface was caused by reduction of lysine residues. Thus, overall, the 2-fold reduction in the proportion of lysine residues in the sequence leads to a 4-fold reduction in the exposed hydrophobic accessible surface area contributed by the associated alkyl component of the lysine side chain.

We noticed that a significant decrease of apolar surface area in the protein synthesized by the halophilic organism can be observed in the OSMOL sample as well, paralleled by an ostensible tendency to increase the presence of side chain oxygen atoms (Table 2). Most of the compatible osmolytes produced by halophilic prokaryotes are neutral species, which contain charged groups (for example amino acid derivatives); therefore, it is not surprising that maintenance of solubility and stability of proteins at decimolar osmolyte concentration, required surface remodeling partly similar to that observed in the SALTIN sample, implying decrease of apolar surface. Indeed, several physical-chemical models have been proposed in the literature, according to which the charged sites of the stabilizing osmolytes interact with the oppositely charged polar areas of the macromolecule surface [25]. On the contrary, stabilizing osmolytes avoid contact with the polypeptide backbone. This property is called "osmophobic effect" [26] and it forces protein to fold in vivo, complementing the well known hydrophobic effect. However, the overall extent of surface modification is much smaller than that observed in the extreme halophilic proteins. Indeed, the examination of the surface atomic potential variations in the SALTIN sample (Table 3) confirms that the electrostatic potential is significantly more negative in halophilic proteins rather in their mesophilic counterparts. Negative surface electrostatic potential has been demonstrated to be a characteristic factor of haloadaptation like, for example, in nucleoside diphosphate kinase from *Halobacterium salinarum *[27] or in an esterase from *Haloarcula marismortui *[19]. Thermal stability of the former protein indeed resulted to be strongly dependent on salt concentration, as predicted by theoretical studies [18]. The picture is different in the case of the OSMOL sample: the increase of the surface formed by side-chain oxygen atoms is not coupled to the decrease of nitrogen atoms: this account for the lack of an apparent modification of surficial potential. Recently, it has been demonstrated [17] by extensive site-directed mutagenesis on three protein domains (the halophilic 1A domain of the NAD-dependent DNA ligase N from *Haloferax volcanii*, the homologous domain from *E.coli*, and the mesophilic IgG binding domain of the protein L from *Streptococcus magnus*) that halophilicity of these proteins is directly related to a decrease in the solvent accessible surface. Authors stated that reduction of the solvent accessible area introduced upon mutation causes a progressive destabilization of the molecule, probably due to a reduction in the protein's hydrophobic effect. As a consequence, mutations increasing salt induced stabilization also destabilize the protein in the absence of salt, converting a mesophilic protein into an obligate halophilic form, a trend found in natural halophilic proteins.

However, we did not observe any significant reduction of the accessibility surface (data not shown) in the SALTIN and OSMOL halophilic proteins whereas we noticed the tendency, in the halophilic proteins of the SALTIN samples, to reduce the apolar contact area of the residues exposing more than 25% of their surface. Therefore one could argue that the reduction of hydrophobic effect be a key component of the haloadaptation mechanisms of surfaces.

However, surface property variations are not the only feature relevant to protein stability and solubility in high salt environments. Indeed, proper folding of the protein in these conditions requires the formation of appropriate hydrophobic interactions in the interior of the macromolecule. This is supposedly even more important during the early step of protein folding of the nascent polypeptide chain. In this perspective view, we analyzed the variations of apolar contact area in the hydrophobic core of the halophilic SALTIN and OSMOL surface compared to their mesophilic counterparts. No statistically significant difference could be found. Nonetheless, it should be noted that a consistent decrease of the apolar contact area in the halophilic proteins of the SALTIN sample can be observed for core residues at 0% relative accessibility (Table 4). We believe this is indicative of a trend, absent in the OSMOL sample, related to haloadaptation of the SALTIN proteins. Similar trend can be observed at the subunit interfaces of extreme halophilic proteins.

Differences in apolar area buried upon folding (*ΔΔ*ApA_U-F_) between each halophilic and non-halophilic pairs display overall no significant difference. This may suggest that, globally, the fraction of apolar area lost during folding is similar in the two cases (halophilic/non-halophilic) although the apolar surface available to the halophilic protein is smaller. This observation is reflected and supported by the significant reduction of exposed apolar area (Table 2) and apolar contact area (Table 4). It should be mentioned however that the difficult definition of unfolded state of a protein [28] should recommend caution in the interpretation of these results. Moreover, in this specific problem, electrostatic repulsion of all the negative charges can render the calculation of the solvent accessible surface area of unfolded state even more inaccurate.

It is well known that high NaCl concentration strengthens hydrophobic interactions [29] and recently the thermodynamics of this effect has been analyzed in synthetic polymers [30]. Hydrophobicity is the main driving force of protein folding [31]; it must be finely balanced to confer proteins proper flexibility and stability compatible with their function. To maintain the magnitude of hydrophobic interactions within useful intervals in a high salt environment, extent of protein interior hydrophobic surface should be reduced in order to lessen the change in solvated hydrophobic areas. Interestingly, it was demonstrated [30] that a potent osmolyte, trimethylamine oxide, have a negligible effect on the strength of hydrophobic interactions. This finding suggests that hydrophobic interactions in proteins of "osmolyte" microorganisms need not to be significantly altered.

**Figure 3:**
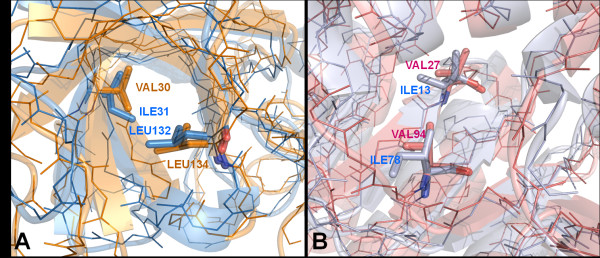
**Examples of residue substitutions decreasing the area of the conserved hydrophobic contacts**. **A) **Superposition of two equivalent CHCs in the halophilic glucose dehydrogenase from *Haloferax mediterranei *(color orange, PDB ID: 2B5W) and its counterpart from *Sulfolobus solfataricus *(color light blue, PDB ID: 2CD9). Secondary structures are represented as cartoon and residues involved in the apolar contact are shown as sticks models. Halophilic Val30 replaces Ile31 of the mesophile. The other contacting residue, Leu, is conserved in both proteins. **B) **Superposition of two equivalent CHCs in the halophilic malate dehydrogenase from *Haloarcula marismortui *(color magenta, PDB ID: 2J5K) and its mesophilic counterpart from *Clostridium thermocellum *(color cyan, PDB ID: 1Y6J). Secondary structures are represented as cartoon and residues involved in the apolar contact are shown as sticks models. Halophilic Val27 and Val94 replace Ile13 and Ile78 of the counterpart, respectively.

This picture is even clearer when inspecting and contrasting the conserved hydrophobic contacts in the proteins belonging to the SALTIN and OSMOL sample. The CHCs [18] are sites in the interior of the protein in correspondence of which there is a hydrophobic contact conserved during divergent evolution. Evolutionary conservation suggests that the contact be essential for folding and/or stability of the protein. The average apolar contact area at the CHCs is significantly smaller in halophilic proteins than in their mesophilic counterparts. As expected, OSMOL sample display no difference (Table 4). Accordingly, the most frequent residue exchange in the SALTIN CHCs is the replacement of Ile with Val in halophilic proteins which reduces the hydrophobic volume buried in the core. Indeed, the effect of such substitution is the loss of a methyl group in the hydrophobic nucleus of the protein. This is expected to reduce the hydrophobic interactions, although conformational strain can also compound the influence on the stability of the protein [32]. As expected, the most frequent residue substitution of the OSMOL CHCs, namely Leu→ Ile, does not imply any reduction in the number of side-chain carbon atoms. As an example, Figure 3 reports two cases taken from the dataset utilized in the work in which a CHC Ile was replaced by Val in the halophilic protein.

## Conclusions

The present analysis suggests that a shared haloadaptation strategy of proteins in the presence of molar salt concentration, but not in the presence of osmolytes, necessitates a weakening of the hydrophobic interactions in general, and in particular at the level of core and conserved hydrophobic contacts. Weakening of these interactions counterbalances their strengthening by the presence of salts in solution and may help the structure preventing aggregation and/or loss of function in hypersaline environments. Indeed, decreasing hydrophobicity makes halophilic proteins unstable in low-concentration salt solutions and may in part explain the request of the halophilic proteins for high salt concentrations. To complete the picture, the destabilization of halophilic proteins at low-salt concentration due to the strong electrostatic repulsion should be considered [18,33]. Shrinking of hydrophobic contacts must be even more critical for the early stages of folding when intramolecular hydrophobic nuclei must correctly form to guide the polypeptide through the folding funnel to the native state.

Considering also the significant increase of biotechnology applications of halophiles, the comprehension of the multifaceted etiology of halophilicity (including the electrostatic factors) can provide the theoretical basis for the engineering of proteins of great interest because stable at concentrations of salts that cause the denaturation or aggregation of the majority of macromolecules.

## Methods

### Selection of protein structures

The crystallographic structures of the available high salt concentration active enzymes were found in PDB [34]. The search was carried out using the keywords: "halophil", haloarc", "halobacter" and the like. For each of the obtained structures, it was verified that the source organism was present in a previously compiled list of halophilic organisms, based on the website XBASE [35]http://www.xbase.ac.uk/ and in the literature. The selected organisms have optimal growth conditions that support the presence of genuine adaptation to high salt concentration environments. In fact, those organisms requiring for optimum growth concentration of NaCl < 0.5 M and/or temperatures < 20°C or > 55°C, were excluded from the data sample. Moreover, the organisms were divided into two groups corresponding to different survival strategies: in the first group are the archaeal halophiles using the "salt-in" strategy (which will be referred to as SALTIN sample); in the latter the *Eubacteria *using the "osmolytes" strategy (OSMOL sample) [36].

To select only halophilic structures matching specific criteria and to eliminate redundancy, we used the PISCES server http://dunbrack.fccc.edu/PISCES.php[37], which enables the creation of a list of structures that meet arbitrary thresholds, starting from an initial user-defined larger sample. The parameters used were: maximum percentage identity 90%, maximum resolution 2.5 Å, maximum R-value 0.3, minimum chain length 50 residues, and utmost chain length 1000 residues. Homologous structures from non-halophilic organisms were retrieved from PDB by means of the program PSI-BLAST [38]. To ensure structural homology, only sequences sharing ≥ 30% and ≤ 90% residue identity to the halophilic counterpart were considered. In cases where more counterparts were available for each halophilic protein, only the homolog with the highest percentage identity to the protein of interest was considered to minimize possible phylogenetic drift effects on the statistics. At the end of the procedure, 15 pairs of halophilic and non-halophilic proteins were collected, 8 of which corresponded to organisms that adopt the "salt-in" strategy and constitute the SALTIN sample, and 7 that adopt the "osmolytes" strategy and compose the OSMOL sample. In cases where the protein structures are used in their biological unit form, the pairs are reduced to 11 because in 4 cases there is no correspondence between the quaternary structure of homologous halophilic and non-halophilic proteins. Although Table 1 shows that the two proteins of the pair 1CNO-1ETP have a different quaternary structure, this pair was included in the analysis. In fact, since 1ETP is a fusion protein of two polypeptide chains each equivalent to the monomer of 1CNO, the two quaternary forms are overall similar.

### Apolar contact area (ACA)

The apolar contact area is the area of contact between coupled non-polar atoms in a protein. It was calculated for different structural environments: nucleus, interface between monomers and protein surface. The program utilized was Pdb_np_cont [39], which calculates the area of contact between coupled non-polar atoms starting from a standard PDB file. Briefly, this method is based on the classification of points located on a sphere of interaction radius, surrounding each non-polar atom. The interaction radius is the van der Waals radius of each atom type, plus the radius of a water molecule. The output of this program was utilized to calculate the pairwise residue contact areas for every possible pair of residues belonging to the structures analysed.

Individual protein chains were used for analysis of the protein core, while analyses of the protein subunit interfaces were carried out with structures in their quaternary assembly. Only the 9 pairs of proteins possessing an oligomeric biological unit were used in this case. Protein interfaces consists of those residues making apolar contacts to another protein chain, as defined by Pdb_np_cont [38].

A residue was assigned to the structural environment "nucleus" if its relative solvent accessibility, calculated with the program NACCESS [40], is less than four different alternative thresholds (0%, 3%, 5% and 9%). A residue is instead assigned to the protein surface if its relative solvent accessibility is greater than an arbitrarily fixed threshold. To this end, four different alternative thresholds (25%, 40%, 55% and 70%) were used.

For each structural environment considered the per-residue mean apolar contact area was calculated by dividing the total apolar contact area by the number of residues belonging to that structural environment.

Conserved hydrophobic contacts (CHCs) were identified in each pair of homologous enzymes through the combined use of the web tools CE-MC [41] for calculating the structural alignment, SCR_FIND for identification of structurally conserved regions and CHC_FIND for the detection of the CHCs and their apolar contact area [42]. SCRs were defined as regions displaying a similar local conformation, with a mean positional RMSD of the equivalent α-carbon positions of the structures superposed ≤ 3.0 Å, lacking indels and composed of at least three consecutive residues. A CHC is defined as a conserved hydrophobic contact formed between two residues belonging to an SCR, in members belonging to a family or superfamily of proteins [42]. We considered only those contacts that are formed between residues distant at least three positions along the sequence and residues that are evolutionarily conserved according to the server CONSURF [43], *i.e*. belonging to the classes 7, 8 and 9. These three classes contain the most conserved amino acid positions from a total of nine equally sized categories of relative degree of conservation.

### Preferred amino acid substitution in CHCs

Amino acid substitutions of residues involved in the formation of structurally and evolutionarily conserved hydrophobic contacts between halophilic and non-halophilic proteins were determined by analysing the alignment of the SCRs of each pair. For each residue *X*, belonging to a non-halophilic protein and involved in making CHCs, *aa*_X→Y _was defined as the number of times *X *is substituted by the residue *Y *of the halophilic protein. Likewise, *aa*_Y→X _is defined. Therefore, a substitution matrix can be obtained by computing the difference between *aa*_X→Y _and *aa*_Y→X _over the whole dataset of protein pairs *k*, according to:

(1) $$C^{S}=\underset{k}{\sum }\left(\sum aa_{X\to Y}-\sum aa_{Y\to X}\right)$$

where *C^S ^*is the element of the substitution matrix.

The mean and standard deviation of the overall exchange matrix were determined; the significance *R_XY _*of the exchange *X→Y *was then calculated by dividing the difference between *C^S^*, and the overall matrix mean $\bar{C}$by the standard deviation σ:

(2) $$R_{XY}=\frac{C^{S}-\bar{C}}{\sigma }$$

*R_XY _*values ≥ 3.0 standard deviations (corresponding to a probability *P *≤ 0.01 that the observed difference was obtained by chance) from the mean value were considered statistically significant [18].

### Accessible surface area (ASA) analysis

The ASA is the area of the protein surface which can be in contact with the solvent. The total apolar (and the complementary polar) component of the entire accessible surface area of each protein in its quaternary form was calculated with the server GETAREA http://curie.utmb.edu/getarea.html with default settings [44]. The contribution of different atomic types to the polar area, namely oxygens, side-chain oxygens, nitrogens and side-chain nitrogens was also investigated. To compare the different surface areas of halophilic and non-halophilic proteins, they were normalized by division by the total accessible surface area of the corresponding protein.

The differences between the fraction of apolar accessible surface area in the unfolded and folded form of each protein considered (*Δ*ApA_U-F_) were calculated through the web server http://roselab.jhu.edu/utils/unfolded.html[28]. The differences between the *Δ*ApA_U-F _in each halophilic protein and in its corresponding homolog (*ΔΔ*ApA_U-F_) were calculated and statistically tested.

### Electrostatic potential calculation

The surface electrostatic potential of the proteins in their quaternary form was calculated using the program DELPHI [45]. Salt concentration was set equal to 0, since identical environmental conditions can better delineate differences between the electrostatic potential of the halophilic and non-halophilic homolog. Internal and solvent dielectric constants were set to 4.0 [46] and 80.0 respectively. The other parameters used were set to the default values: grid scale = 1.2, box fill = 60%, probe radius = 1.4 Å, and van der Waals surface. To compare the potential of halophilic and non-halophilic proteins of different lengths, the average atomic potential (AAP) was calculated dividing the total electrostatic potential by the total number of atoms.

### Statistical tests

Whenever possible, differences between the structural properties considered were calculated within the two samples: the SALTIN halophilic and non-halophilic homologs, and the OSMOL halophilic and non-halophilic pairs. Differences of structural properties are denoted by *Δ*: for example, *Δ*ACA indicates halophilic minus non-halophilic apolar contact areas.

The *Δ*s between the structural properties of halophilic enzymes and their corresponding non-halophilic counterparts were tested within samples using two statistical tests, a paired *t*-test and a non-parametric Wilcoxon signed-rank test [47]. In the former case the null hypothesis is that the average *Δ *was 0 at 0.05 *p*-value while in the latter case the null hypothesis is that the median of the *Δ*s was 0 at 0.05 *p*-value. The parametric *t*-test assumes that the tested data come from a normal distribution, while the Wilcoxon test it is less restrictive since it does not require such a condition. To enhance robustness of the conclusions drawn from the structural comparisons, only differences resulting significant from both tests were deemed *bona fide *significant results.

## List of abbreviations

*Δ*: prefix indicating the difference between the property measured in the halophilic and in the non-halophilic homologous proteins; AAP: average atomic potential; ACA: apolar contact area; ApA: apolar accessible area; *Δ*ApA_U-F_: difference between ApA in the unfolded and folded state; ASA: solvent accessible surface area; CHC: conserved hydrophobic contacts; OSMOL: halophilic organisms adopting the "osmolytes" strategy; RMSD: Root mean square deviation; SALTIN: halophilic organisms adopting the "salt-in" strategy; SCR: structurally conserved region.

## Authors' contributions

AS and MP collected the data, carried out the calculations and analysed results. AP conceived the study, helped to analyse results and to correct the manuscript. SP analysed the results, drafted the manuscript and participated in study design and coordination. All authors read and approved the final manuscript and declare no conflict of interest.

## Supplementary Material

Additional file 1 **Table S1 - *Δ*ASA in the SALTIN and OSMOL samples at conserved residues**. Differences of fractional accessibility surface area (*Δ*ASA) in the SALTIN and OSMOL samples for different class of atoms. The differences are between the surface areas calculated in the halophilic protein and the corresponding areas in the non-halophilic counterpart. The calculations were performed considering the proteins in their quaternary structure. Only residues identically conserved in the two proteins were considered in the calculations. Click here for file

Additional file 2 **Additional Figure 1 - *ΔΔ*ApA_U-F_**. Histograms reporting the *ΔΔ*ApAU-F in the SALTIN and OSMOL samples. The *ΔΔ*ApA_U-F _values were calculated as the difference between the fraction of exposed apolar area lost during folding of the halophilic protein and the fraction lost by the corresponding non-halophilic homolog. Further details are reported in the "Methods" section of the main text. Click here for file

Additional file 3 **Additional Figure 2 - Correlation between single *Δ*ACA and pairwise percentage identity**. Graph reporting the difference between the area of each halophilic CHC and that of the corresponding non-halophilic CHC (*Δ*ACA) *versus *the pairwise sequence percentage identity (%id) for the SALTIN and OSMOL samples. Click here for file
