# Supplementary material for: Structural adaptation of extreme halophilic proteins through decrease of conserved hydrophobic contact surface
Source: BMC Struct Biol. 2011 Dec 22;11:50. doi: 10.1186/1472-6807-11-50 (PMC3293032; doi:10.1186/1472-6807-11-50)
Supplement: Additional file 1 — Table S1 - ΔASA in the SALTIN and OSMOL samples at conserved residues. Differences of fractional accessibility surface area (ΔASA) in the SALTIN and OSMOL samples for different class of atoms. The differences are between the surface areas calculated in the halophilic protein and the corresponding areas in the non-halophilic counterpart. The calculations were performed considering the proteins in their quaternary structure. Only residues identically conserved in the two proteins were considered in the calculations. [file 1472-6807-11-50-S1.DOC]

**Table S1 - *Δ*ASA in the SALTIN and OSMOL samples at conserved residues**

| SALTIN  HALOPHILES | NON  HALOPHILES | *Δ*ApA^a)^ | *Δ*Tot O^b)^ | *Δ*Sc O^c)^ | *Δ*Tot N^d)^ | |  | *Δ*Sc N^e)^ | OSMOL  HALOPHILES | NON  HALOPHILES | *Δ*ApA^a)^ | *Δ*Tot O^b)^ | *Δ*Sc O^c)^ | *Δ*Tot N^d)^ | *Δ*Sc N^e)^ |
| --- | --- | --- | --- | --- | --- | --- | --- | --- | --- | --- | --- | --- | --- | --- | --- |
| 1DOI | 1FXA | -0.11 | -0.07 | -0.06 |  | 0.00 |  | 0.00 | 1NWZ | 1MZU | -0.03 | 0.00 | 0.00 | -0.02 | -0.02 |
| 1TJO | 2VXX | -0.18 | -0.04 | -0.01 |  | -0.03 |  | -0.02 | 3IBM | 3KGZ | 0.02 | 0.05 | 0.05 | -0.01 | -0.02 |
| 2CC6 | 2V18 | 0.06 | 0.04 | 0.03 |  | 0.00 |  | -0.01 | 1CNO | 1ETP | 0.01 | 0.01 | 0.01 | 0.01 | 0.01 |
| 1ITK | 2FXG | 0.00 | 0.01 | 0.01 |  | 0.00 |  | 0.00 | 2VPN | 3FXB | -0.04 | -0.01 | 0.00 | -0.01 | -0.01 |
| 2AZ3 | 3B54 | 0.05 | 0.06 | 0.06 |  | 0.00 |  | 0.00 | 3BSM | 2QJJ | 0.11 | 0.03 | 0.02 | 0.05 | 0.06 |
| 3IFV | 1RWZ | -0.05 | -0.03 | -0.02 |  | -0.04 |  | -0.03 |  |  |  |  |  |  |  |
| Total^f)^ | | -0.23 | -0.03 | 0.01 |  | -0.06 |  | -0.05 |  |  | 0.07 | 0.08 | 0.08 | 0.03 | 0.01 |
| Average^g)^ | | -0.04 | 0.00 | 0.00 |  | -0.01 |  | -0.01 |  |  | 0.01 | 0.02 | 0.02 | 0.01 | 0.00 |
| t-test | | 0.35 | 0.82 | 0.93 |  | 0.24 |  | 0.15 |  |  | 0.63 | 0.18 | 0.18 | 0.64 | 0.85 |

Differences of fractional accessibility surface area (*Δ*ASA) in the SALTIN and OSMOL samples for different class of atoms. The differences are between the surface areas calculated in the halophilic protein and the corresponding areas in the non-halophilic counterpart. The calculations were performed considering the proteins in their quaternary structure. Only residues identically conserved in the two proteins were considered in the calculations.

a) apolar *Δ*ASA difference between fractional apolar exposed areas of the halophilic protein and the corresponding non–halophilic homolog

b) oxygen atom fractional *Δ*ASA

c) sidechain oxygen atom fractional *Δ*ASA

d) nitrogen atom fractional *Δ*ASA

e) sidechain nitrogen fractional *Δ*ASA

f) Total fractional *Δ*ASA

g) Average fractional *Δ*ASA
