# Supplementary material for: Structural adaptation of extreme halophilic proteins through decrease of conserved hydrophobic contact surface
Source: BMC Struct Biol. 2011 Dec 22;11:50. doi: 10.1186/1472-6807-11-50 (PMC3293032; doi:10.1186/1472-6807-11-50)
Supplement: Additional file 2 — Additional Figure 1 - ΔΔApAU-F. Histograms reporting the ΔΔApAU-F in the SALTIN and OSMOL samples. The ΔΔApAU-F values were calculated as the difference between the fraction of exposed apolar area lost during folding of the halophilic protein and the fraction lost by the corresponding non-halophilic homolog. Further details are reported in the "Methods" section of the main text. [file 1472-6807-11-50-S2.DOC]

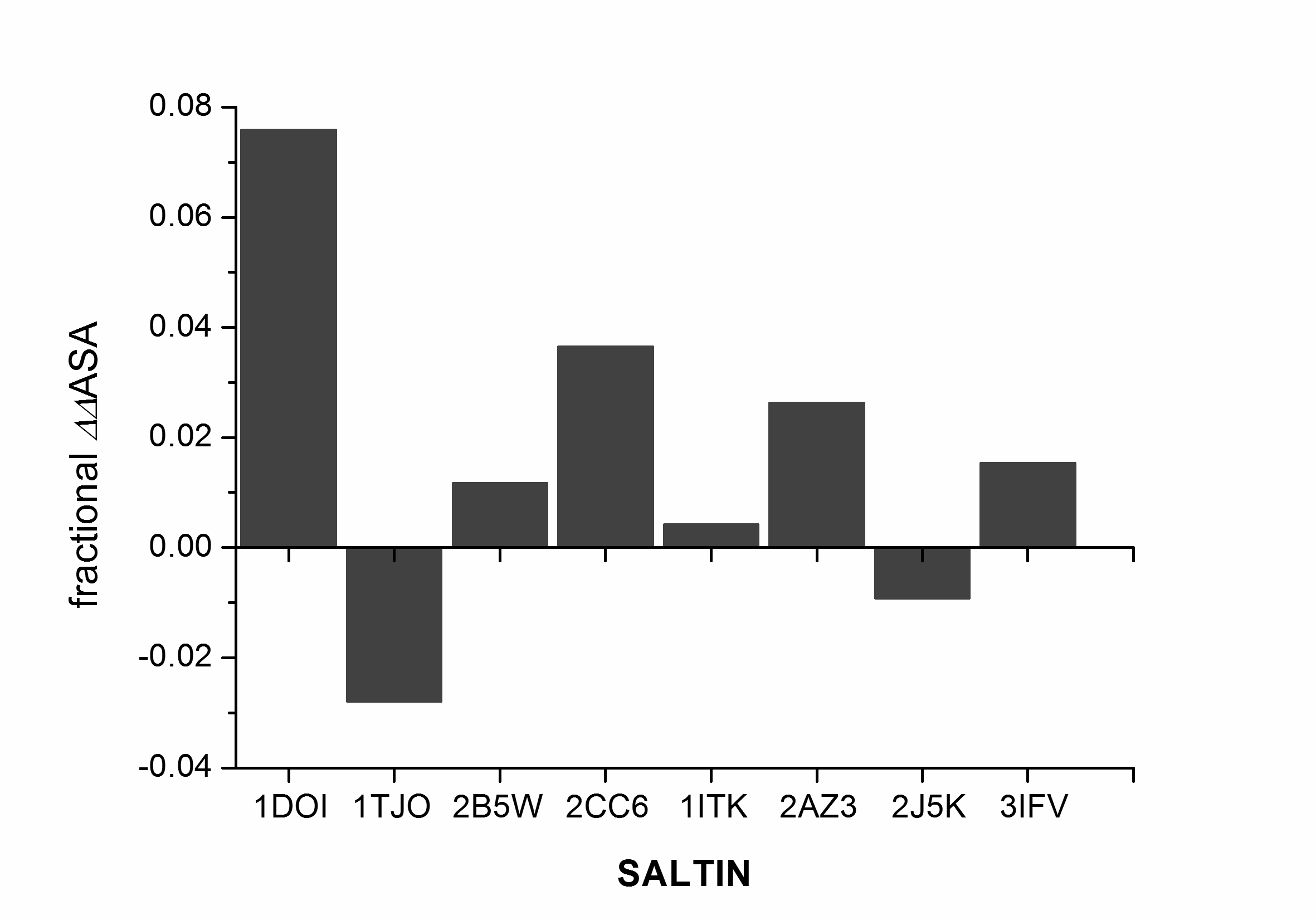


Figur


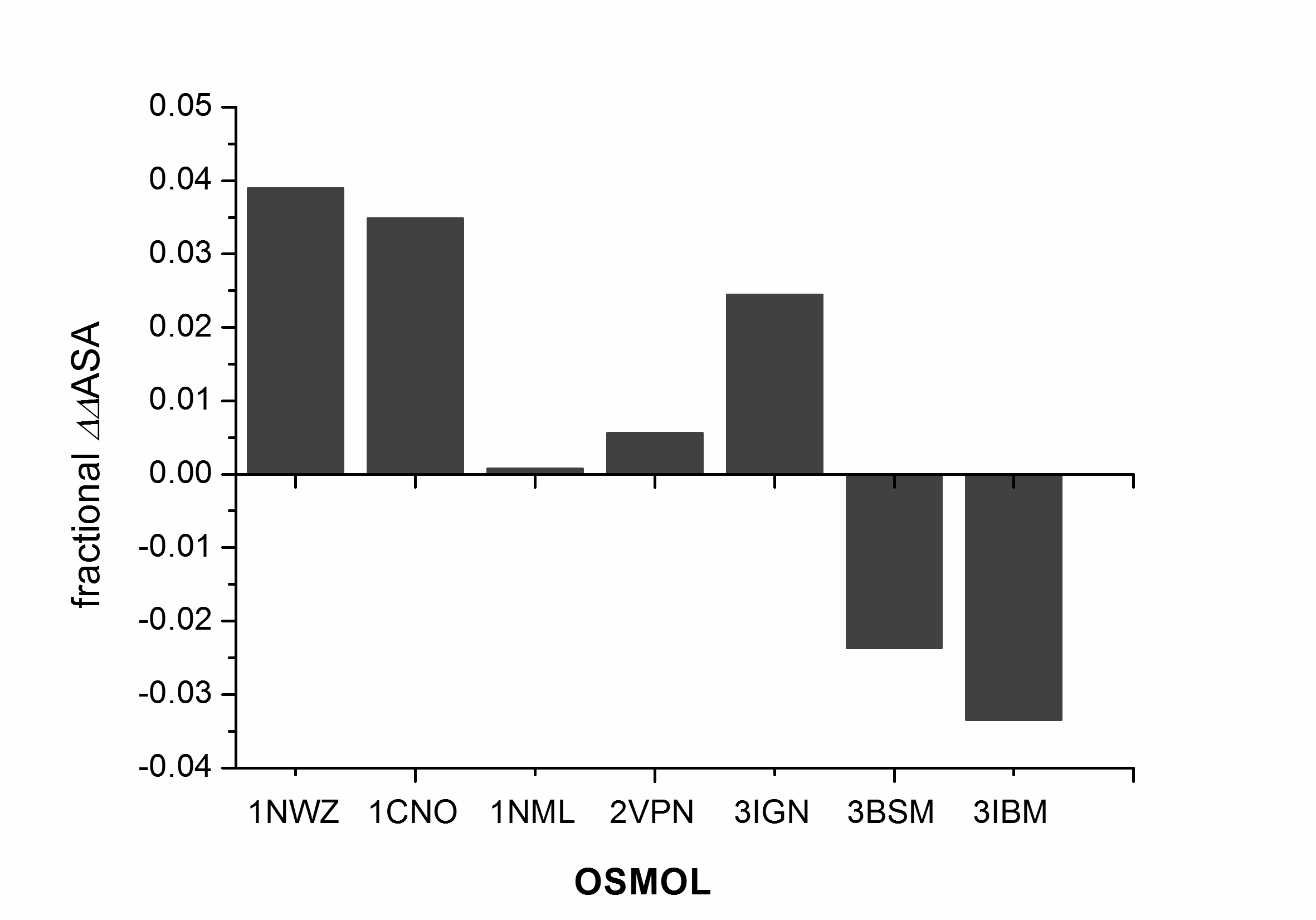


**Additional Figure 1 - *ΔΔ*ApA_U-F_**

Histograms reporting the *ΔΔ*ApA_U-F_ in the SALTIN (upper panel) and OSMOL (lower panel) samples. The *ΔΔ*ApA_U-F_ values were calculated as the difference between the fraction of exposed apolar area lost during folding of the halophilic protein and the fraction lost by the corresponding non-halophilc homolog. Further details are reported in the “Methods” section. PDB codes on the *x*-axis indicate the halophilic protein. In both cases, average differences, tested with the Wilcoxon and the *t*-test, were not significantly different from 0.
