# Supplementary material for: Structural adaptation of extreme halophilic proteins through decrease of conserved hydrophobic contact surface
Source: BMC Struct Biol. 2011 Dec 22;11:50. doi: 10.1186/1472-6807-11-50 (PMC3293032; doi:10.1186/1472-6807-11-50)
Supplement: Additional file 3 — Additional Figure 2 - Correlation between single ΔACA and pairwise percentage identity. Graph reporting the difference between the area of each halophilic CHC and that of the corresponding non-halophilic CHC (ΔACA) versus the pairwise sequence percentage identity (%id) for the SALTIN and OSMOL samples. [file 1472-6807-11-50-S3.DOC]

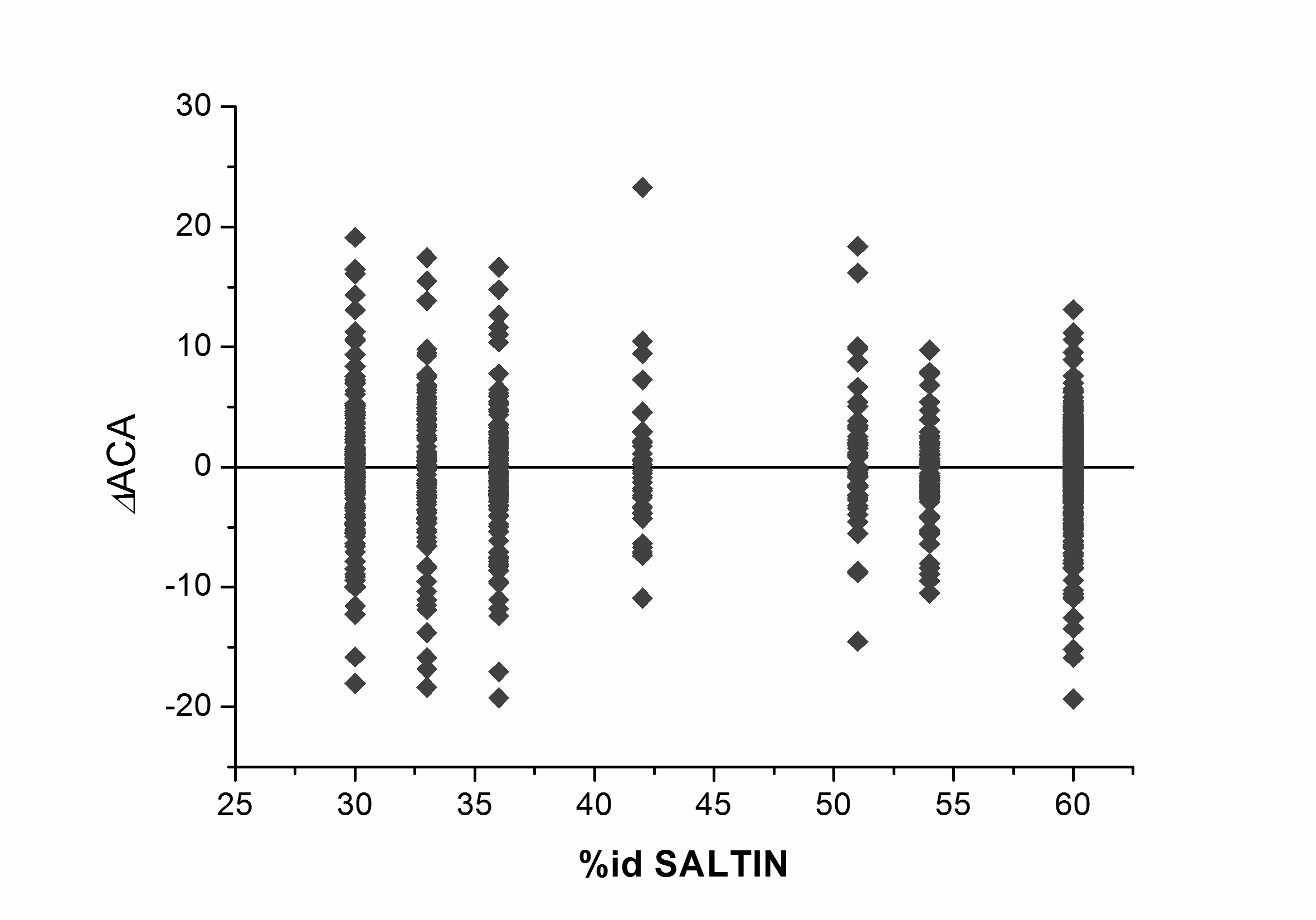


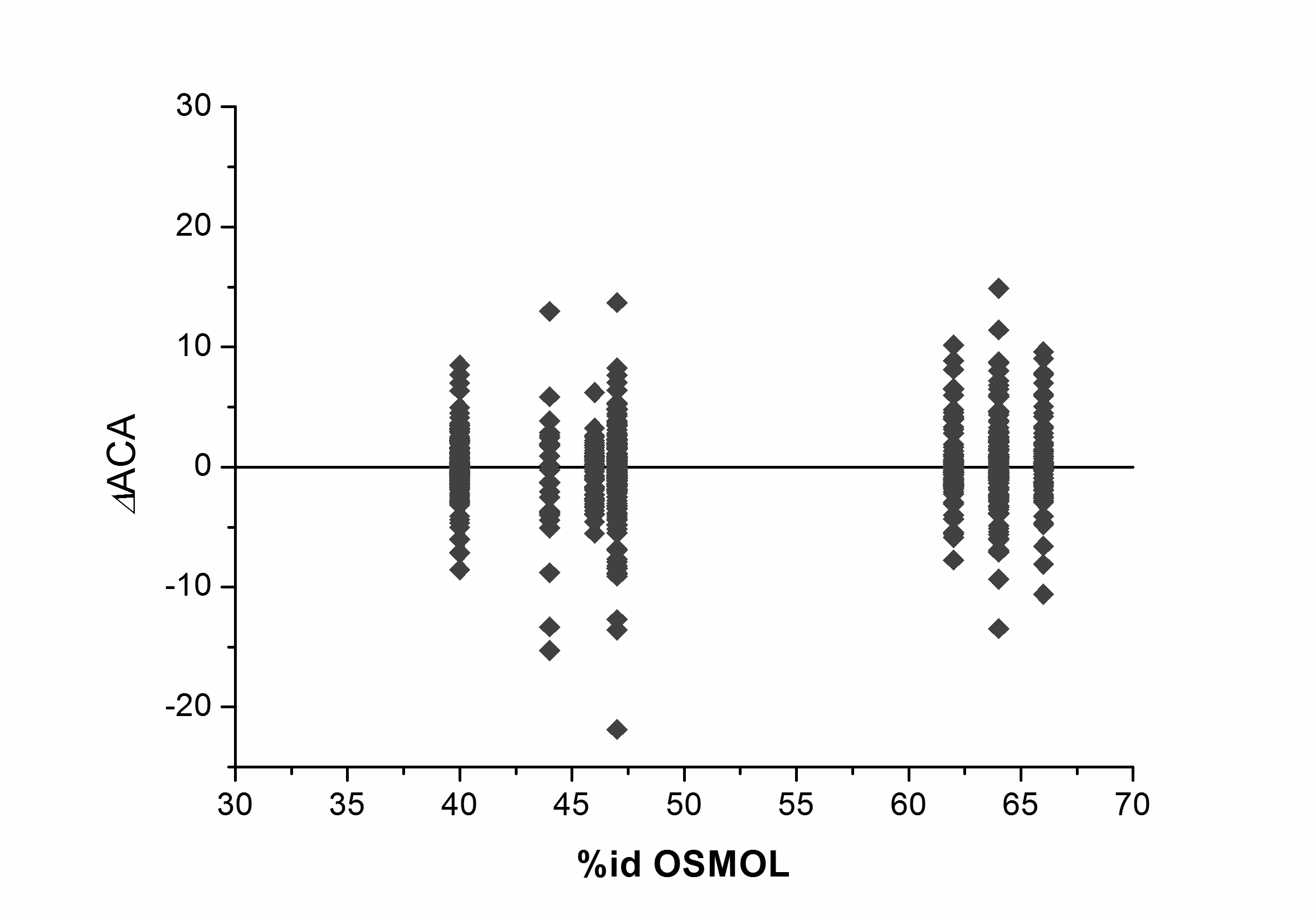


**Additional Figure 2 – Correlation between single *Δ*ACA and pairwise percentage identity.**

The difference between the area of each halophilic CHC and that of the corresponding non-halophilic CHC (*Δ*ACA) is reported *versus* the pairwise sequence percentage identity (%id) for the SALTIN and OSMOL samples.
